# Transcriptome sequencing of *Pinus kesiya var. langbianensis* and comparative analysis in the *Pinus* phylogeny

**DOI:** 10.1186/s12864-018-5127-6

**Published:** 2018-10-03

**Authors:** You-jie Zhao, Yong Cao, Juan Wang, Zhi Xiong

**Affiliations:** 1 Key Laboratory for Forest Resources Conservation and Utilization in the Southwest Mountains of China, Ministry of Education, Southwest Forestry University, Kunming, 650224 Yunnan People’s Republic of China; 2 College of Big data and Intelligent Engineering, Southwest Forestry University, Kunming, 650224 Yunnan People’s Republic of China; 3 Eco-development Academy, Southwest Forestry University, Kunming, 650224 Yunnan People’s Republic of China; 4 College of Light industry and Food, Southwest Forestry University, Kunming, 650224 Yunnan People’s Republic of China

**Keywords:** *Pinus kesiya* var. *langbianensis*, Transcriptome sequencing, *Pinus* phylogeny, Comparative transcriptomics

## Abstract

**Background:**

Pines are widely distributed in the Northern Hemisphere and have a long evolutionary history. The availability of transcriptome data has facilitated comparative transcriptomics for studying the evolutionary patterns associated with the different geographical distributions of species in the *Pinus* phylogeny.

**Results:**

The transcriptome of *Pinus kesiya* var. *langbianensis* was sequenced using the Illumina HiSeq 2000 platform, and a total of 68,881 unigenes were assembled by Trinity. Transcriptome sequences of another 12 conifer species were downloaded from public databases. All of the pairwise orthologues were identified by comparative transcriptome analysis in 13 conifer species, from which the rate of diversification was calculated and a phylogenetic tree inferred. All of the fast-evolving positive selection sequences were identified, and some salt-, drought-, and abscisic acid-resistance genes were discovered.

**Conclusions:**

mRNA sequences of *P. kesiya* var*. langbianensis* were obtained by transcriptome sequencing, and a large number of simple sequence repeat and short nucleotide polymorphism loci were detected. These data can be used in molecular marker-assisted selected in pine breeding. Divergence times were estimated in the 13 conifer species using comparative transcriptomic analysis. A number of positive selection genes were found to be related to environmental factors. Salt- and abscisic acid-related genes exhibited different selection patterns between coastal and inland *Pinus*. Our findings help elucidate speciation patterns in the *Pinus* lineage.

**Electronic supplementary material:**

The online version of this article (10.1186/s12864-018-5127-6) contains supplementary material, which is available to authorized users.

## Background

Pines (*Pinus*) are widely distributed in the Northern Hemisphere and are the largest extant genus of conifers, constituting the most important source of wood in the forestry industry [1, 2]. The 115 species of *Pinus* are divided into two subgenera [3] and possess a rich fossil record dating back as much as 130–140 million years ago (Mya) [4, 5]. Many studies have focused on this genus, particularly with regards to its phylogenetic relationships [6, 7] and the timing of diversification events [6, 8–10]. However, no study has examined how pines adapted to their varied ecological environments over evolutionary history.

Transcriptome sequencing technology can obtain all of the RNA information of an organism at a point in time, thereby providing a large amount of information for molecular biology studies [11–13]. In particular, Illumina SOLEXA sequencing has been widely used for the excavation and discovery of functional genes. Transcriptome sequencing can also obtain a large number of single nucleotide polymorphism (SNP), simple sequence repeat (SSR), and other molecular markers [14, 15]. Molecular markers with good polymorphism can help researchers shorten the genetic breeding cycle [16].

As increasing numbers of species have been included in transcriptome sequencing projects, comparative transcriptomics has received greater attention from researchers [9, 17–19]. Comparative transcriptomics can elucidate the phylogenetic relationships of multiple species and can assess the functional differences between orthologous genes following divergence. The functional differences between orthologous genes constitute important evidence for studying patterns of evolution in different *Pinus* species from different geographical regions. *Pinus* species have adapted to different habitats, including low-latitude and high-latitude environments, and coastal and inland areas (Table 3). In Eurasia, *P. sylvestris* is mainly distributed in inland and high-latitude regions, whereas *P. pinea*, *P. halepensis*, and *P. pinaster* are coastal species distributed in the Mediterranean region and southern Europe. In North America, *P. banksiana* is distributed in the inland and high-latitude areas of North American and Canada, whereas *P. taeda* and *P. palustris* occur in the coastal areas of the southeastern United States. In Asia, *P. tabuliformis* is an inland and high-latitude *Pinus* species that occurs mainly in northern China.

*Pinus kesiya* var. *langbianensis* (*P. kesiya* var. *langbianensis*) is a coastal species in Asia, and it is mainly distributed in southwest China and southeast Asia. In this study, we performed transcriptome sequencing of *P. kesiya* var. *langbianensis* using the Illumina HiSeq2000 platform. Following sequence assembling and analysis, unigenes and some molecular markers in *P. kesiya* var. *langbianensis* were obtained. Comparative transcriptomics was subsequently used in 13 conifer species. A number of positive selection genes were determined to be related to environmental factors in the *Pinus* species from different geographical regions.

## Results

### Transcriptome sequencing and de novo assembly

The cDNA samples obtained from pine needles of *P. kesiya* var. *langbianensis* and other individuals were normalized to increase the sequencing efficiency of rare transcripts. A total of about 141 million reads with an average insert size of 200 bp were generated from the Illumina HiSeq 2000 platform. After cleaning and quality control, about 133 million high-quality reads were obtained with a total length of 11.99 Gb (Table 1). Cleaned reads were de novo assembled to 68,881 unigenes with a mean size of 821 bp, representing a total length of about 42.5 Mb and a mean size of 713 bp. More than half of the total assembly length of the unigenes was > 1402 bp (N50 = 1402). The coverage distribution revealed that most unigenes had a read-depth coverage of 20-fold, and 29% unigenes were up to 100-fold (Additional file 1: Figure S1).

**Table 1:** Transcriptome sequencing and assembly of *P. kesiya var. langbianensis* transcriptome

Sequencing results		Assembly results	
Total number of raw reads	141,234,042	Total number of unigenes	68,881
Total number of clean reads	133,236,316	Total unigenes length (bp)	56,537,842
Total clean Nucleotides (bp)	11,991,268,440	Contig N50 (bp)	1402
Q20 (%)	98.25%	Mean length (bp)	821
N content(%)	0.01%	Max length (bp)	15,498
GC content (%)	45.02%	Min length (bp)	200

### Functional annotation of the transcriptome

**Fig. 1:**
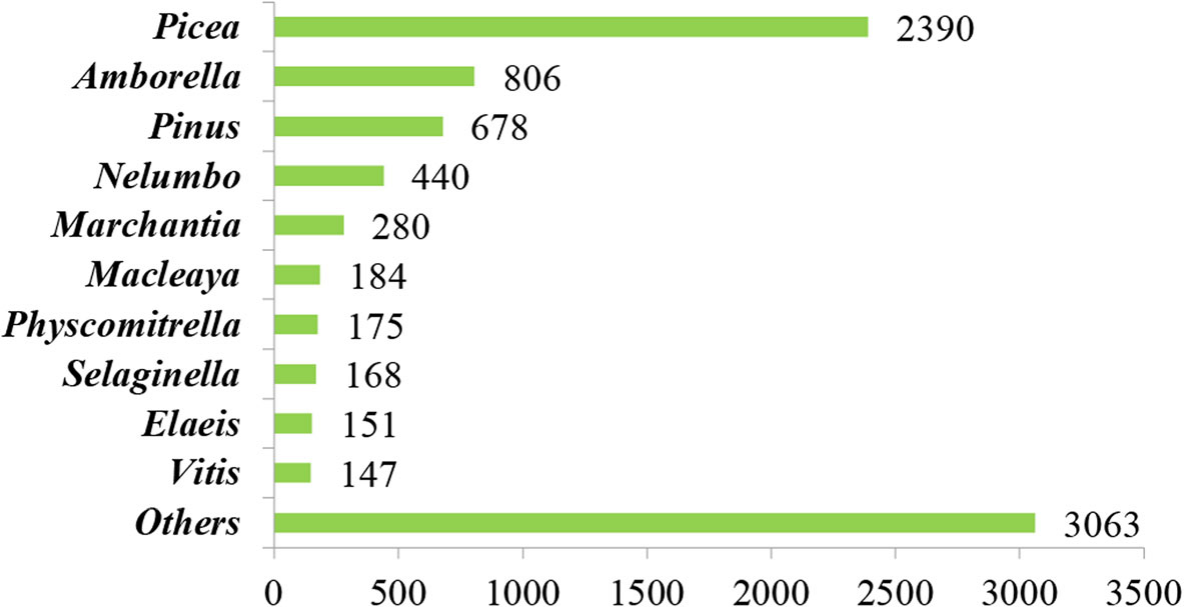
Summary and taxonomic source of the BLASTX matches to unigenes. Number of unique best BLASTX matches of unigenes grouped by genus

A total of 48,035 (69.74%) *P. kesiya* var. *langbianensis* unigenes were significantly matched to known genes in the public databases (Table 2). Approximately 37,757 unigenes were aligned to the NCBI Non-redundant (Nr) protein database. We examined the taxonomic distribution of the BLASTX best hits group by genus. As a result, only 8482 unigenes had specific functional annotations, 28.18% were within *Picea*, and 7.99% were within *Pinus* (Fig. 1). About 23,491 unigenes (34.10%) were assigned to 42 functional groups based on Gene Ontology (GO) assignments (Additional file 2: Table S1), including biochemistry, growth, development, metabolism, apoptosis, and immune defense.

**Table 2:** Unigene annotation of *P. kesiya var. langbianensis* transcriptome

Unigenes	NR	NT	Swiss-Prot	KEGG	COG	GO	ALL
68,881	37,757	42,869	24,093	21,433	13,528	23,491	48,035

### Identification of SSRs and SNPs

**Fig. 2:**
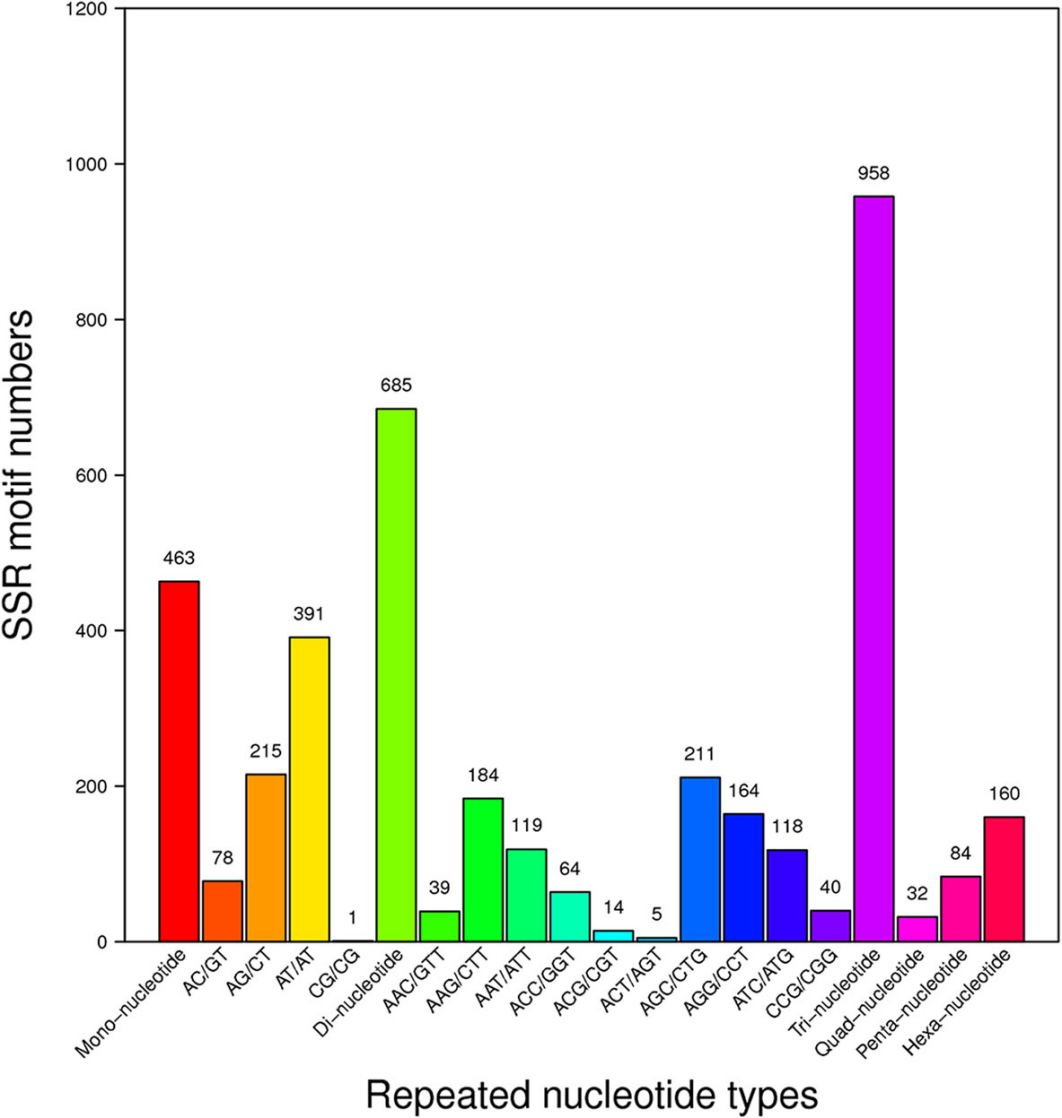
Distribution of SSRs in the *P. kesiya* var. *langbianensis* transcriptome

**Fig. 3:**
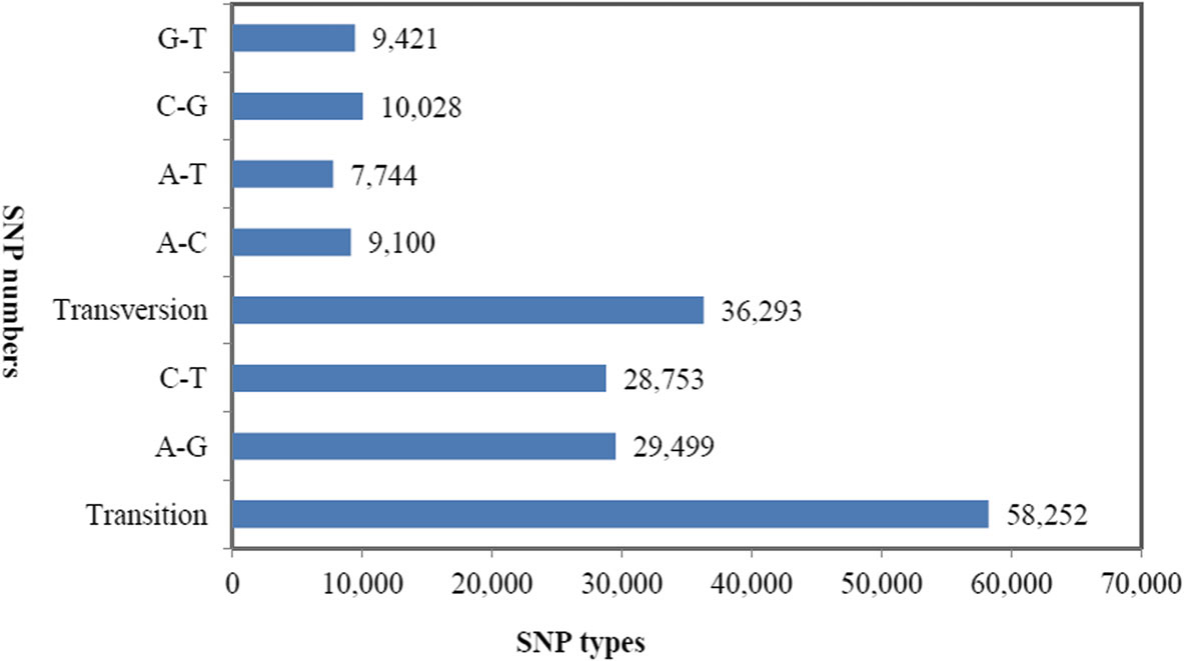
Distribution of SNPs in the *P. kesiya* var. *langbianensis* transcriptome

A total of 2382 distinct loci were identified, and the incidences of different repeat types were determined (Fig. 2). Among the different classes of SSRs, the tri-nucleotide repeats were the most abundant (40.22%), followed by di-nucleotides (28.76%). Similar results were found in other conifer species, with the exception of *P. pinaster* (Additional file 2: Table S2). More than 94,545 SNPs were identified from the *P. kesiya* var. *langbianensis* transcriptome (Fig. 3). Among all of the SNPs, transitions (61.61%) were more frequent than transversions (38.39%). A and G were the most frequent SNPs (31.20%), and A and G were the second-most frequent (30.41%).

### Orthologue identification and functional characterization of 13 conifer species

**Fig. 4:**
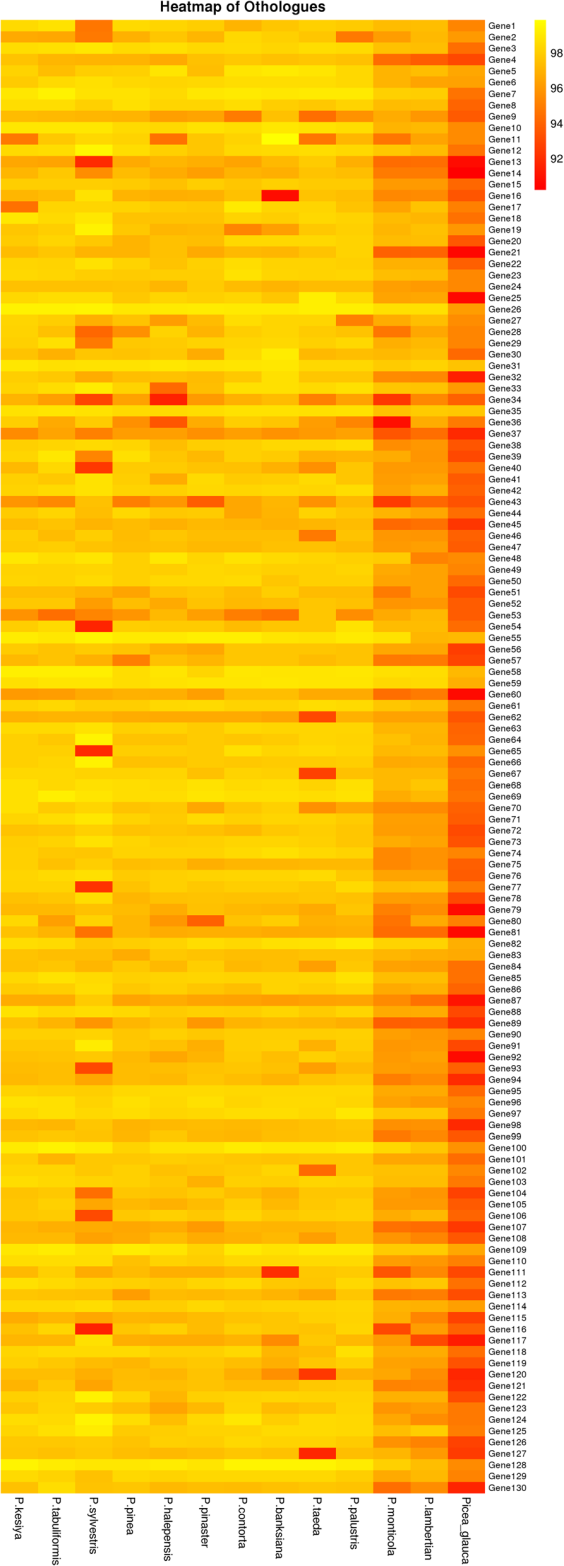
Functional annotation and divergence between the orthologues of 12 pine and one spruce species. The heat map is based on the 130 putative orthologous transcripts of the 13 species. The orthologues were annotated to different functions based on GO terms (Additional file 3: File S1). Colors indicate similarity from yellow (highly similar) to red (weakly similar)

Public transcriptome sequences were downloaded from the PlantGDB (*http://www.plantgdb.org**/*) and NCBI databases (Table 3). The average number of unigenes was about 42,335 in the 13 conifer species, and *P. taeda* had the largest number of unigenes (91,046), while *P. tabuliformis* had the least (10,285). The average unigene length mostly exceeded 656 bp, except for *P. sylvestris* and *P. monticola*. All of the pairwise orthologues were identified by comparative analysis between the 13 conifer species (Table 4). The results showed that *P. taeda* had the maximum average number (7571) of orthologous genes, whereas *P. sylvestris* had the minimum average number (1552). The highest number of orthologous genes (16,051) was found between *P. kesiya* var. *langbianensis* and *P. halepensis*, while the lowest number (727) was found between *P. sylvestris* and *P. halepensis*. One hundred and thirty shared orthologues were found in all 13 conifer species (Fig. 4). The orthologues were annotated with GO terms (Additional file 2: Table S3), and 119 orthologues were involved in biological processes, cellular components, and molecular functions, and the other 11 orthologues had unknown biological functions.

**Table 3:** Transcriptome sequencing in 13 conifer species

*Pinus/Picea* spp.	Main distribution	Data source	Number of Unigenes	Total length(bp)	Mean length(bp)
*P. kesiya*	Southwestern China southeastern Asian	Illumina Sequencing	68,881	58,975,225	856
*P. tabuliformis*	Northern China northern Korea	NCBI SRA(SRR546476)	10,285	8,669,639	843
*P. sylvestris*	Middle and high latitudes of Eurasia	PlantGDB	73,609	29,552,311	401
*P. pinea*	Mediterranean region southern Europe	NCBI SRA(SRR445497, SRR445498)	11,403	10,122,178	888
*P. halepensis*	Mediterranean region	NCBI SRA(SRR942848)	72,028	47,224,748	656
*P. pinaster*	Mediterranean region	PlantGDB	15,648	11,498,176	735
*P. contorta*	Western North America	PlantGDB	13,570	15,334,600	1130
*P. banksiana*	North American Canada	PlantGDB	13,040	14,756,117	1132
*P. taeda*	Southeastern United States	Treegenesdb (v2.01)	91,046	75,408,866	828
*P. palustris*	Southeastern United States	NCBI SRA(SRR065012)	15,013	14,111,464	940
*P. monticola*	Mountains of western United States and Canada	NCBI SRA(SRR1013836)	86,230	33,420,814	388
*P. lambertian*	Mountains of western North America	NCBI SRA(SRR064207)	30,981	33,041,789	1067
*Picea glauca*	Northern United States southern/central Canada	PlantGDB	48,619	54,962,881	1130

**Table 4:** Number and Ks peaks of orthologous genes in 13 conifer species

	*P. kesiya*	*P. tabuliformis*	*P. sylvestris*	*P. pinea*	*P. halepensis*	*P. pinaster*	*P. contorta*	*P. banksiana*	*P. taeda*	*P. palustris*	*P. monticola*	*P. lambertian*	*Picea glauca*
*P. kesiya*													
*P. tabuliformis*	6351/0.01												
*P. sylvestris*	4331/0.01	898/0.01											
*P. pinea*	8112/0.03	4144/0.03	1153/0.02										
*P. halepensis*	16,051/0.03	5498/0.03	727/0.02	5839/0.03									
*P. pinaster*	7561/0.02	3311/0.03	944/0.02	3844/0.03	6419/0.03								
*P. contorta*	8922/0.03	3953/0.03	1129/0.03	4392/0.04	6578/0.04	4011/0.03							
*P. banksiana*	7784/0.03	3577/0.03	1374/0.03	3815/0.04	5642/0.04	3610/0.04	4996/0.01						
*P. taeda*	13,493/0.04	4981/0.03	2624/0.03	6114/0.04	14,088/0.04	6496/0.04	6836/0.01	5744/0.01					
*P. palustris*	9311/0.03	3514/0.03	1092/0.03	4097/0.04	8425/0.04	4035/0.04	4321/0.01	3994/0.01	7551/0.01				
*P. monticola*	7472/0.08	3538/0.08	1433/0.06	4481/0.08	5940/0.08	3790/0.09	4773/0.07	4769/0.06	4847/0.09	5087/0.08			
*P. lambertian*	11,185/0.08	4477/0.08	1964/0.06	5233/0.08	8269/0.09	4783/0.08	5308/0.08	4722/0.08	8349/0.08	6428/0.08	7169/0.01		
*Picea glauca*	13,691/0.14	4808/0.15	959/0.14	6493/0.15	12,041/0.15	5082/0.15	7999/0.14	7092/0.14	9724/0.15	6019/0.14	7710/0.15	9969/0.15

### Phylogenetic and divergence time analysis

**Fig. 5:**
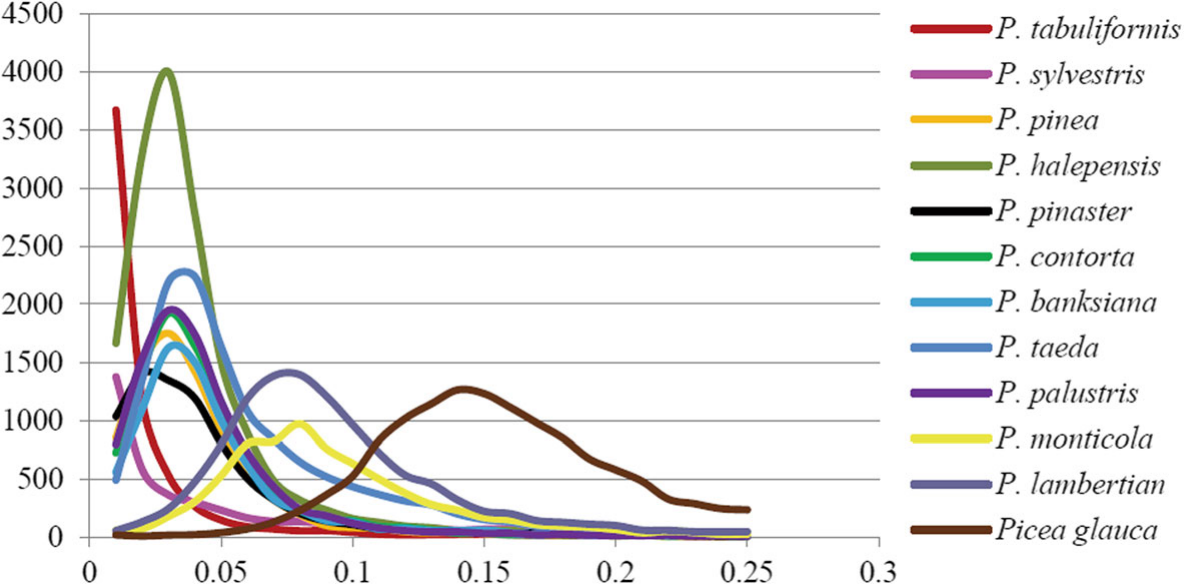
Distribution of Ks values of the orthologous pairs between *P. kesiya* var. *langbianensis* and others

The genetic distance of species is related to the synonymous mutation rate calculated by the orthologous genes, and thus the synonymous mutation rate was estimated for all of the pairs of orthologues in the 13 conifer species (Table 4). *Pinus kesiya* var. *langbianensis* and *P. taeda* yielded 13,493 orthologues with the synonymous substitutions per synonymous site (Ks) values exhibiting a normal distribution with a peak value of 0.04. The minimum Ks peak was detected in *P. kesiya* var. *langbianensis* (< 0.01) with *P. tabuliformis* and *P. sylvestris*, followed by a 0.03 Ks peak with *P. banksiana* and *P. contorta*, and a 0.08 Ks peak with *P. monticola* and *P. lambertiana*, and the maximum Ks peak of 0.15 with the outgroup *Picea glauca* (Fig. 5). The relationship reflected by the Ks distance of *P. kesiya* var. *langbianensis* corroborated the *Pinus* phylogeny [6, 7]. As observed in previous studies of six conifer species [9], *P. tabuliformis* had the same Ks peak of 0.03, 0.03, and 0.01 with *P. taeda*, *P. contorta*, and *P. sylvestris*, respectively.

**Fig. 6:**
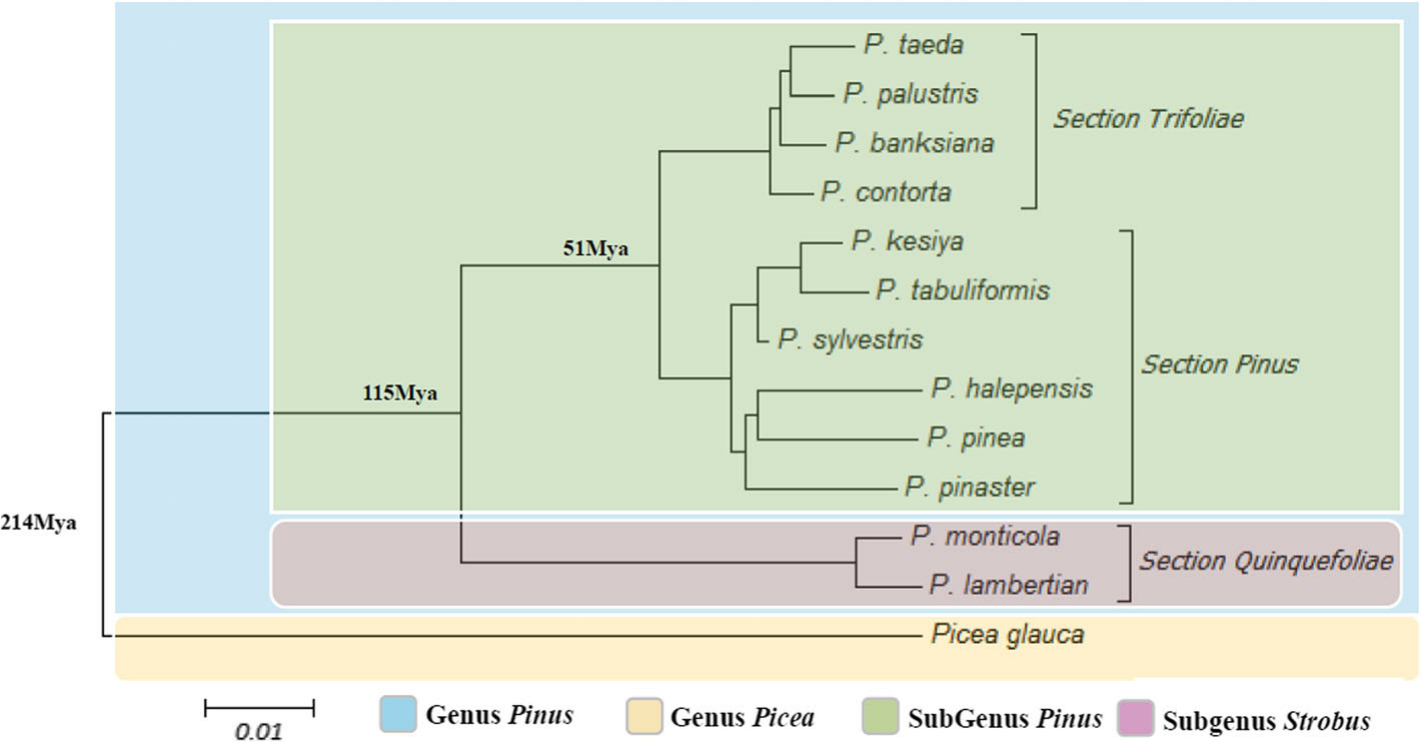
Phylogenetic tree of 12 *Pinus* and one *Picea* species. The phylogram was derived using the pairwise synonymous substitution rates of the orthologous transcripts as a distance metric and the neighbor-joining method

Using *Picea glauca* as an outgroup species, the phylogenetic tree of *Pinus* was constructed based on the Ks peak distance matrix (Fig. 6). The phylogenetic relationships were highly consistent with the phylogenetic tree obtained from cpDNA and mtDNA analyses in previous studies [6, 7]. In the phylogenetic tree, the average Ks value was 0.146 between *Pinus* and *Picea*; 0.078 between subgenus *Strobus* and subgenus *Pinus*; and 0.035 between section *Trifoliae* and section *Pinus*. In the previous study of *Picea sitchensis* and *P. taeda*, it was calculated that the synonymous mutation rate of the molecular clock in genus *Pinus* was about 0.68*10^− 9^ substitutions/site/year, and the divergence time could be expressed as Ks*10^− 6^/0.68*10^− 9^. Thus, the divergence time was estimated at about 214 Mya (Triassic) between *Pinus* and *Picea*; about 115 Mya (Cretaceous) between subgenus *Strobus* and subgenus *Pinus*; and about 51 Mya (Paleogene) between section *Trifoliae* and section *Pinus*.

### Evolutionary pattern of *Pinus* spp. genes

The number of pairwise synonymous (Ks) and non-synonymous (Ka) substitutions per site can reflect the evolutionary patterns of species. *Ka/Ks* > 1 indicates that the gene has been involved in positive selection during evolution (Table 5). A number of positive selection genes were found to be related to environmental factors (Table 6).

**Table 5:** Number and contained function of positive selection genes in Genus *Pinus*

	*P.kesiya*	*P.tabuliformis*	*P.sylvestris*	*P.pinea*	*P.halepensis*	*P.pinaster*	*P.contorta*	*P.banksiana*	*P.taeda*	*P.palustris*	*P.monticola*	*P.lambertian*
*P.kesiya*												
*P.tabuliformis*	2750/AS											
*P.sylvestris*	1700/D	422										
*P.pinea*	949	505	267									
*P.halepensis*	2173	720/A	154	697								
*P.pinaster*	1267	561	227	573	1032							
*P.contorta*	1169	519	299	411	720	511						
*P.banksiana*	960/A	453	338	361/A	603/A	444	1685/A					
*P.taeda*	1823/S	648	697	512	1798	887	1685/S	1386/AS				
*P.palustris*	1101	444/A	268	323	754	497	1335	1110/A	2839			
*P.monticola*	271	189	135	177	209	195	235	266/A	209	215		
*P.lambertian*	398	154/A	200	122	221	205	156	166	296	204	2688/D	

*A* Abscisic acid stress, *D* Drought stress, *S* Salt stress

**Table 6:** Information of resistance genes involved in positive selection in Genus *Pinus*

Funtion	Nr Annotation	Specie1	Gene1	Specie2	Gene2	Ka/Ks	Ka	Ks
Salt stress	NP_001105589.1	*P. tabuliformis*	isotig03867	*P. kesiya*	CL1082.Contig1_All	> 1	0.0013	0
Salt stress	NP_001105589.1	*P. taeda*	PITA_000000350-RA	*P. banksiana*	PUT-177a-Pinus_banksiana-8213	4.3893	0.0873	0.0199
Salt stress	NP_001105589.1	*P. taeda*	PITA_000000350-RA	*P. kesiya*	CL1082.Contig1_All	4.9134	0.0994	0.0202
Salt stress	NP_001105589.1	*P. taeda*	PITA_000000350-RA	*P. contorta*	PUT-175a-Pinus_contorta-5514	4.3893	0.0873	0.0199
Drought stress	AAD37375.1	*P. sylvestris*	PUT-187a-Pinus_sylvestris-52,858	*P. kesiya*	Unigene23170_All	1.0047	0.0083	0.0083
Drought stress	AAD37375.1	*P. lambertian*	isotig24007	*P. monticola*	comp65821_c3	> 1	0.0046	0
ABA stress	NP_001105719.1	*P. palustris*	isotig03955	*P. banksiana*	PUT-177a-Pinus_banksiana-10,254	1.2072	0.0054	0.0045
ABA stress	NP_001105719.1	*P. kesiya*	Unigene11406_All	*P. banksiana*	PUT-177a-Pinus_banksiana-10,253	1.166	0.0262	0.0224
ABA stress	NP_001105719.1	*P. contorta*	PUT-175a-Pinus_contorta-11,400	*P. banksiana*	PUT-177a-Pinus_banksiana-10,253	1.2	0.0162	0.0135
ABA stress	NP_001105719.1	*P. taeda*	PITA_000045763-RA	*P. banksiana*	PUT-177a-Pinus_banksiana-10,254	> 1	0.004	0
ABA stress	NP_001105719.1	*P. pinea*	isotig05514	*P. banksiana*	PUT-177a-Pinus_banksiana-10,253	1.0145	0.05	0.0493
ABA stress	NP_001105719.1	*P. halepensis*	comp18337_c1	*P. banksiana*	PUT-177a-Pinus_banksiana-10,253	1.2039	0.0601	0.0499

ABA is Abscisic acid. Sequences showed in Additional file 4: File S2

In section *Pinus*, 2750 positive selection genes were found between *P. tabuliformis* and *P. kesiya* var. *langbianensis*, including one salt stress-related gene (Nr annotation: NP_001105589.1) via the production of the NADP-dependent protein [20, 21]. In section *Trifoliae*, *P. taeda* shared 1386 and 1685 positive selection genes with *P. banksiana* and *P. contorta*, including the same salt stress-related gene (Nr annotation: NP_001105589.1). Furthermore, 1700 positive selection genes were identified between *P. kesiya* var*. langbianensis* and *P. sylvestris*, including a drought stress-related gene (Nr annotation: AAD37375.1) via the production of the peroxidase protein [22, 23]. In section *Quinquefoliae*, 2688 positive selection genes were discovered between *P. lambertiana* and *P. monticola*, including one drought stress-related gene (Nr annotation: AAD37375.1). In genus *Pinus*, *P. banksiana* shared 361, 603, 960, 1685, 1386, 1110, and 266 positive selection genes with *P. pinea*, *P. halepensis*, *P. kesiya* var. *langbianensis*, *P. contorta*, *P. taeda*, and *P. palustris*, and one gene related to abscisic acid stress (Nr annotation: NP_001104929.1) was commonly found in all of the pairs.

The results indicate that salt and abscisic acid stress genes were obviously involved in positive selection between coastal and inland *Pinus* species.

## Discussion

### Paleogeographic events in the divergence of the *Pinus* phylogeny

*Pinus* and *Picea* diverged about 214 Mya at the end of the Triassic (252–201 Mya). Compared with previous studies [8, 9], the time between *Pinus* and *Picea* was older than the previous result of 174–190 Mya. At the beginning of the Triassic, only one supercontinent Pangea was present on earth, and the climate was singly warm and dry [24, 25]. The ancient continent was rifting during the late of Triassic with the climate becoming more diverse, during which *Pinus* and *Picea* diverged.

The divergence time of the subgenera *Strobus* and *Pinus* was about 115 Mya, in the middle of the Cretaceous (145–66 Mya). Compared with previous studies, the time was between the previous results of 85 Mya and 128 Mya [6, 10]. During the Cretaceous period, the distribution of the land and sea changed rapidly as a result of tectonic activity in the Atlantic Ocean, and large-scale transgression occurred in the coastal areas (a transgressive event) [26]. The species of subgenus *Strobus* were mainly distributed in the coastal mountains of western North America. In the middle of Cretaceous period, broad shallow seas advanced across central North America. This transgressive event might reflect the geographical speciation of subgenus *Strobus* and shown to be consistent with the divergence time 115 Mya in our results.

The divergence time of sections *Pinus* and *Trifoliae* was about 51 Mya during the Paleogene (66–23 Mya) [27]. There was a dispute over this time in previous studies [6, 8–10]. One suggests the divergence time of section *Pinus* and section *Trifoliae* was occurred in the beginning of Paleogene, and another suggests it was in the Late Cretaceous (~ 100–66 Ma). Our result supports the former conclusion, but older than the former’s result of 44–45 Mya [8, 9]. The divergence of sections *Pinus* and *Trifoliae* may be related to the separation of Laurasia [28] into Eurasia and Laurentia during the Paleocene (66–56 Mya) which is the first epoch of Paleogene.

### Salt stress gene selection between coastal and inland *Pinus*

Paleogeography (http://www.scotese.com) shows the southern areas of North America had been covered by shallow water during the Paleogene. *P. taeda* is mainly distributed in the coastal areas of southeastern US, while *P. banksiana* is an inland *Pinus* species in the North America and Canada. The regressive event altered the habitat of *P. taeda* from shallow sea into land, which may explain the involvement of the salt stress gene in the selective evolution between *P. taeda* and inland *P. banksiana*. It might also explain the positive selection of the salt stress gene between coastal *P. kesiya* var. *langbianensis* (southwest China) and *P. tabuliformis* (inland of Northern China).

### Abscisic acid stress gene selection between coastal and inland *Pinus*

By 50 Mya, the global climate departed from the hot and humid conditions and began a cooling and drying trend towards a series of ice age [29]. Most species of sections *Pinus* and *Trifoliae* had gone through this cool stage especially the high-latitude. Production of abscisic acid is increased by cold and drought and acts to help plants withstand these conditions [30–32]. *Pinus banksiana* occurs in the high-latitude inland regions of North America and Canada, which is possibly related to the positive selection of the abscisic acid gene in this species in comparison to the coastally distributed species *P. taeda* (southeastern US)*, P. palustris* (southeastern US), and *P. kesiya* var. *langbianensis* (southwestern China, Southeast Asia).

## Conclusions

In the present study, the phylograms and divergence times were estimated by comparative transcriptomic analysis in 13 conifer species. A number of positive selection genes were found to be associated with environmental factors. The divergence times suggest that plate movement and transgression events caused the geographical speciation which might be the key drives in the divergence of *Pinus* phylogeny. The analysis of selection evolution suggests salt and abscisic acid-related genes were involved in positive selection between coastal and inland *Pinus* species. These data are useful for evaluating the different evolutionary patterns between inland and coastal *Pinus* species. The study shows that tectonic plate movement, and transgression and regression events resulted in changes to the land and sea, and adaptive evolution may have played an important role in the divergence of the *Pinus* species.

## Methods

### RNA extraction and sequencing

Pine needle tissue, including the cambium, of *P. kesiya* var. *langbianensis* was collected from Puer City, Yunnan Province, China. The specimen was identified by Yong-chun Xu and Juan Wang, whose deposition numbers are 0000651 and 0000652 from the herbarium of Southwest Forestry University. Total RNA was isolated using RNAiso Plus (TaKaRa, Japan). RNA quality was characterized initially on an agarose gel and NanoDrop ND1000 spectrophotometer (NanoDrop Technologies, Wilmington, DE, USA).

Illumina sequencing based on a GAII platform was performed at the Beijing Genomics Institute (Shenzhen, China; http://www.genomics.cn), following the manufacturer’s protocols. A fragmentation buffer was added to interrupt the mRNA and thereby generate fragments in the size of range 200 bp. The resulting fragments served as a template for the synthesis of the first-strand cDNA, employing random hexamer primers (N6). Second-strand cDNA was synthesized using a SuperScript Double-Stranded cDNA Synthesis kit (Invitrogen, Camarillo, CA), after which it was purified using a QiaQuick PCR extraction kit (Qiagen, Hilden, Germany) and resolved with EB buffer for end repair and poly (A) addition. The products were ligated with one another using sequencing adapters, and a suitable fragment size range was selected for PCR amplification following agarose gel electrophoresis. The resulting library was sequenced using an Illumina HiSeqTM 2000 platform.

### Data filtering and de novo assembly

Image data output from the sequencing device were transformed into raw reads and stored in FASTQ format. These data were filtered to remove raw reads that included adapter sequences or those that were of low quality. Transcriptome assembly was achieved using the short-read assembly program Trinity [33]. The unigenes were divided into either clusters or singletons. BLASTX [34] alignment between each unigene sequence and those lodged in the Nr, Nt (Nucleotide database, NCBI), Swiss-Prot, GO (http://www.geneontology.org/), and COG (clusters of orthologous groups) databases were performed, and the best alignments were used to infer the directionality of the unigene. Where the outcomes from the various databases conflicted with one another, the priority order applied was: Nr, Swiss-Prot, and COG. Where no alignment was possible, the software tool ESTScan [35] was used to assign directionality.

### Gene annotation and analysis

Functional annotation was assigned using the protein (Nr and Swiss-Prot), COG, and GO databases. BLASTX was employed to identify related sequences in the protein databases. The COG database attempts to classify proteins from completely sequenced genomes on the basis of the orthology concept [36]. The aim of GO is to standardize the representation of genes and their products by insisting on a controlled vocabulary and a strictly defined concept [37, 38]. The annotations acquired from Nr were processed through the Blast2GO program [39] to obtain the relevant GO terms, and these were then analyzed by WEGO software [40] to assign a GO functional classification and to illustrate the distribution of the gene functions.

### Identification of SSRs and SNPs

Unigenes containing putative SSRs and SNPs were identified by MISA and SOAPsnp [41] software. Mono- to hexa-nucleotide SSRs with a minimum repeat unit size of 12 (for mono-), 6 (for di-), 5 (for tri- and tetra-), and 4 (for penta- and hexa-) were identified based on the analysis of the assembled *P. kesiya* unigenes.

### Identification of orthologues between the 13 conifer species

In order to discover the evolutionary patterns of orthologues in *Pinus*, transcriptome sequences of 11 *Pinus* and *Picea glauca* (outgroup) accessions were downloaded from the PlantGDB and NCBI databases (Table 3). *Pinus banksiana*, *P. contorta*, *P. sylvestris*, *P. pinaster*, and *Picea glauca* were directly derived from PlantGDB. *Pinus taeda* was obtained from the NCBI genome database [42, 43]. *Pinus monticola*, *P. lambertiana*, *P. pinea*, *P. halepensis*, *P. tabuliformis*, and *P. palustris* were obtained from the NCBI SRA database. The 454 RNA dataset was assembled using Newbler (http://roche-applied-science.com/) software, and the Illumina RNA dataset was assembled by Trinity software. The assembled sequences were combined and clustered with CD-HIT (version 4.0) [44, 45]. Sequences with similarity > 95% were divided into one class, and the longest sequence of each class was treated as a unigene during later processing.

The transcribed sequences were clustered using UCLUST software [46]. Aligned sequences showing 90% identity were defined as pairs of putative orthologues among the 13 species. The best-hit sequence of each cluster was then used in the subsequent analyses.

### Estimation at the synonymous substitution and non-synonymous substitution levels between orthologues

Since unigenes are derived from expressed sequence tag (EST) sequences, have no annotated open reading frames, and may contain frame shift sequencing errors, each member of a pair of sequences was searched using BLASTX against all of the plant protein sequences available in GenBank. The approach used was as described previously [47]. PAML (http://abacus.gene.ucl.ac.uk/software/paml.html) software was used to estimate the non-synonymous substitutions per non-synonymous site (Ka) and the synonymous substitutions per synonymous site (Ks) [48].

### Phylogenetic analysis

As the phylogenetic relationships in *Pinus* are well understood [6, 7], the precise topology is not critical for the purposes of this study. We chose to focus our analyses on the evolutionary patterns and rate of genetic divergence. The synonymous substitutions and non-synonymous substitutions between the orthologues of the 13 conifer species were analyzed as described previously. Phylograms were derived using the pairwise Ks values of the orthologous transcripts as a distance metric based on the neighbor-joining method [49]. *Picea glauca* was used as an outgroup to root the trees. The distance of two branches was estimated based on the average of all of the pairwise Ks values in two branches of the phylogenetic tree.

## Additional files

Additional file 1: **Figure S1.** Distribution of unigene length and depth. (TIFF 7129 kb)

Additional file 2: **Table S1.** Gene Ontology (GO) distributions for *P. kesiya var. langbianensis*. **Table S2.** SSR markers identified in 13 conifer species. **Table S3.** Gene Ontology (GO) distributions for shared orthologues in 13 conifer species. (XLSX 16 kb)

Additional file 3: **File S1.** Go annotation of 130 shared unigenes (PDF 17 kb)

Additional file 4: **File S2.** Sequences of resistance unigenes (PDF 14 kb)

## Abbreviations

COG: Clusters of orthologous groups
EST: Expressed sequence tag
GO: Gene Ontology
Ka: Non-synonymous substitutions per non-synonymous site
Ks: Synonymous substitutions per synonymous site
Mya: Million years ago
Nr: NCBI Non-redundant protein database
SNP: Single nucleotide polymorphism
SSR: Simple sequence repeat
