# Supplementary material for: Transcriptome sequencing of Pinus kesiya var. langbianensis and comparative analysis in the Pinus phylogeny
Source: BMC Genomics. 2018 Oct 3;19:725. doi: 10.1186/s12864-018-5127-6 (PMC6171231; doi:10.1186/s12864-018-5127-6)
Supplement: Supplementary file 3 — File S1. Go annotation of 130 shared unigenes (PDF 17 kb) [file 12864_2018_5127_MOESM3_ESM.pdf]

Gene id,GO Annotation
Gene1,GO:0000038|GO:0051287|GO:0006633|GO:0042335|GO:0055114|GO:0016747|GO:0005739|GO:0009416|GO:0009409|GO:0009611|GO:0009913|GO:0016616|GO:0009579|GO:0008863|GO:0009507|GO:0046686|GO:0080167|GO:0016020|GO:0005783|GO:0005634
Gene2,GO:0000502|GO:0009651
Gene3,GO:0000724|GO:0009507
Gene4,GO:0000786|GO:0003677|GO:0006334|GO:0005634|GO:0046982
Gene5,GO:0001510|GO:0006412|GO:0005618|GO:0005730|GO:0009506|GO:0003735|GO:0022627|GO:0009507|GO:0005794|GO:0005886
Gene6,GO:0003712|GO:0006357|GO:0043565|GO:0003700|GO:0046982|GO:0005634
Gene7,GO:0003735|GO:0003723|GO:0015935|GO:0006412
Gene8,GO:0003735|GO:0005886|GO:0005618|GO:0022625|GO:0009507|GO:0006412
Gene9,GO:0003735|GO:0042254|GO:0022625|GO:0016020|GO:0006412
Gene10,GO:0003743|GO:0006413|GO:0003746|GO:0006452|GO:0034050|GO:0042742|GO:0009611|GO:0008612|GO:0045905|GO:0043022|GO:0010089|GO:0045901|GO:0046686|GO:0005634
Gene11,GO:0003743|GO:0006413|GO:0005829|GO:0005730|GO:0008026|GO:0009505|GO:0005524|GO:0046686|GO:0005886
Gene12,GO:0004069|GO:0051788|GO:0006635|GO:0009536|GO:0043161|GO:0009693|GO:0009407|GO:0080129|GO:0005618|GO:0048767|GO:0005777|GO:0005829|GO:0080130|GO:0006099|GO:0006531|GO:0030170|GO:0010150|GO:0006536|GO:0006103|GO:0009735|GO:0005886|GO:0005507|GO:0006522|GO:0005739
Gene13,GO:0004553|GO:0006073|GO:0048046|GO:0005618|GO:0016762
Gene14,GO:0005488|GO:0016621|GO:0019761|GO:0009809|GO:0005829
Gene15,GO:0005506|GO:0016226|GO:0005739|GO:0051536
Gene16,GO:0005507|GO:0004013|GO:0006730|GO:0016441|GO:0000166|GO:0006346|GO:0005773|GO:0005886
Gene17,GO:0005507|GO:0004478|GO:0006556|GO:0006730|GO:0005524|GO:0005737|GO:0005886
Gene18,GO:0005507|GO:0005730|GO:0009651|GO:0009409|GO:0003735|GO:0006414|GO:0042254|GO:0010043|GO:0009507|GO:0046686|GO:0022626|GO:0005886
Gene19,GO:0005507|GO:0009651|GO:0009416|GO:0009409|GO:0000015|GO:0009737|GO:0000287|GO:0006096|GO:0005740|GO:0009507|GO:0046686|GO:0004634|GO:0005634|GO:0005886|GO:0048046
Gene20,GO:0005576|GO:0016491|GO:0005634|GO:0016209|GO:0010027|GO:0055114|GO:0009507
Gene21,GO:0005618|GO:0000786|GO:0005730|GO:0009294|GO:0009611|GO:0009617|GO:0003677|GO:0006334|GO:0046982
Gene22,GO:0005618|GO:0031072|GO:0006457|GO:0005730|GO:0009651|GO:0043462|GO:0046872|GO:0009408|GO:0005524|GO:0051082|GO:0005886
Gene23,GO:0005618|GO:0042744|GO:0055114|GO:0005739|GO:0050897|GO:0009416|GO:0009409|GO:0004096|GO:0009941|GO:0009737|GO:0009570|GO:0020037|GO:0009970|GO:0005773|GO:0006995|GO:0046686|GO:0022626|GO:0016036|GO:0009514|GO:0005886|GO:0048046
Gene24,GO:0005634|GO:0016925|GO:0031386|GO:0009408
Gene25,GO:0005730|GO:0003735|GO:0005886|GO:0003723|GO:0005618|GO:0022627|GO:0009507|GO:0006412
Gene26,GO:0005730|GO:0005773|GO:0003735|GO:0009506|GO:0005618|GO:0022627|GO:0016020|

GO:0006412
Gene27,GO:0005730|GO:0005773|GO:0005886|GO:0005618|GO:0022625|GO:0009507
Gene28,GO:0005730|GO:0005773|GO:0005886|GO:0005618|GO:0022625|GO:0009507
Gene29,GO:0005730|GO:0022626|GO:0009644|GO:0009409|GO:0006979|GO:0003735|GO:0005618|GO:0016020|GO:0006412|GO:0000166
Gene30,GO:0005737|GO:0005730|GO:0042273|GO:0006413|GO:0042256|GO:0003743|GO:0043023|GO:0043022
Gene31,GO:0005737|GO:0005886|GO:0016567|GO:0006511|GO:0004842|GO:0005634
Gene32,GO:0005737|GO:0045548|GO:0009800|GO:0006559
Gene33,GO:0005763|GO:0003676|GO:0046686|GO:0003735|GO:0022627|GO:0005634|GO:0006412|GO:0009651
Gene34,GO:0005763|GO:0003676|GO:0046686|GO:0003735|GO:0022627|GO:0005634|GO:0006412|GO:0009651
Gene35,GO:0005773|GO:0005525|GO:0005886|GO:0016787|GO:0005634|GO:0007264|GO:0015031
Gene36,GO:0005773|GO:0005886|GO:0006950|GO:0005618|GO:0005747|GO:0016021|GO:0009507
Gene37,GO:0005773|GO:0010200|GO:0006865
Gene38,GO:0005774|GO:0048046|GO:0004550|GO:0006241|GO:0009651|GO:0009507|GO:0005794|GO:0005777|GO:0005829|GO:0006165|GO:0006183|GO:0006228|GO:0046686|GO:0009506|GO:0005886|GO:0005524
Gene39,GO:0005794|GO:0006810|GO:0005215|GO:0031348
Gene40,GO:0005794|GO:0009055|GO:0045454|GO:0008794|GO:0009507|GO:0015035|GO:0005783
Gene41,GO:0005794|GO:0016023|GO:0005886|GO:0004190|GO:0016021|GO:0006508|GO:0010008
Gene42,GO:0005829|GO:0005507|GO:0005618|GO:0005730|GO:0009651|GO:0006096|GO:0005740|GO:0004332|GO:0009507|GO:0046686|GO:0080167|GO:0005886|GO:0048046
Gene43,GO:0005829|GO:0005507|GO:0005618|GO:0055114|GO:0006164|GO:0005739|GO:0009651|GO:0009941|GO:0004455|GO:0009097|GO:0009099|GO:0000287|GO:0070402|GO:0006096|GO:0009570|GO:0016853|GO:0006094|GO:0042803|GO:0046686|GO:0048046
Gene44,GO:0005829|GO:0005507|GO:0009086|GO:0008705|GO:0008270|GO:0005777|GO:0009651|GO:0009941|GO:0003871|GO:0032259|GO:0009570|GO:0010043|GO:0046686|GO:0005886|GO:0048046
Gene45,GO:0005829|GO:0005634|GO:0005509
Gene46,GO:0005829|GO:0042744|GO:0055114|GO:0005739|GO:0016021|GO:0046872|GO:0009506|GO:0009941|GO:0016688|GO:0005778|GO:0005774|GO:0020037|GO:0005794|GO:0005886
Gene47,GO:0005829|GO:0055114|GO:0046872|GO:0043090|GO:0016616|GO:0005524|GO:0006499|GO:0006888|GO:0048037
Gene48,GO:0005886
Gene49,GO:0005886|GO:0008295|GO:0004766
Gene50,GO:0005975|GO:0008810
Gene51,GO:0006007|GO:0000786|GO:0003677|GO:0006605|GO:0006334|GO:0046982|GO:0005634
Gene52,GO:0006094|GO:0009941|GO:1901671|GO:0048046|GO:0006457|GO:0019288|GO:0009651|GO:0005829|GO:0006096|GO:0009570|GO:0009658|GO:0046686|GO:0009409|GO:0019344|GO:0009535|GO:0005507|GO:0005516|GO:0005524|GO:0048481|GO:0005739
Gene53,GO:0006399|GO:0009941|GO:0008270|GO:0035304|GO:0002103|GO:0010207|GO:0009902|GO:0009965|GO:0004842|GO:0030154|GO:0045893|GO:0042793|GO:0045037|GO:0010027|GO:000

5739

Gene54,GO:0006412|GO:0005618|GO:0005730|GO:0006364|GO:0003735|GO:0022627|GO:0030686|GO:0042274|GO:0032040|GO:0009507|GO:0005886

Gene55,GO:0006412|GO:0005739|GO:0005730|GO:0003735|GO:0009507|GO:0022625|GO:0009793

Gene56,GO:0006412|GO:0005840|GO:0006098|GO:0003735|GO:0009507

Gene57,GO:0006412|GO:0009955|GO:0005730|GO:0003735|GO:0009965|GO:0010015|GO:0009507|GO:0008283|GO:0005773|GO:0022625|GO:0005886|GO:0008097

Gene58,GO:0006413|GO:0003743

Gene59,GO:0006413|GO:0003743|GO:0016070|GO:0016020|GO:0005739

Gene60,GO:0006536|GO:0030170|GO:0046686|GO:0004351|GO:0016706|GO:0016702|GO:0009536|GO:0005516|GO:0055114

Gene61,GO:0006544|GO:0010197|GO:0032259|GO:0035999|GO:0009555|GO:0004372|GO:0006563|GO:0046686|GO:0030170|GO:0008168|GO:0005886

Gene62,GO:0006635|GO:0004557|GO:0006891|GO:0016023|GO:0009505|GO:0009311|GO:0005773|GO:0006869|GO:0010351|GO:0016558|GO:0009755|GO:0048767|GO:0005576|GO:0046477|GO:0016139

Gene63,GO:0006944|GO:0009504|GO:0009506|GO:0005886|GO:0008565|GO:0016192|GO:0009507

Gene64,GO:0007275

Gene65,GO:0008143|GO:0005634|GO:0000166|GO:0005886

Gene66,GO:0008171|GO:0032259

Gene67,GO:0008234|GO:0005829

Gene68,GO:0008270

Gene69,GO:0008270

Gene70,GO:0008299|GO:0004421

Gene71,GO:0008641|GO:0046686|GO:0005886|GO:0016567|GO:0005524|GO:0004842|GO:0051707

Gene72,GO:0009250|GO:0009011

Gene73,GO:0009407|GO:0005634|GO:0005886

Gene74,GO:0009507|GO:0005773

Gene75,GO:0009522|GO:0006098|GO:0035304|GO:0030003|GO:0010207|GO:0009507|GO:0070838|GO:0019344|GO:0009657|GO:0009768|GO:0006364|GO:0005739

Gene76,GO:0009536

Gene77,GO:0009651

Gene78,GO:0009651|GO:0016558|GO:0016023|GO:0006635|GO:0006816|GO:0007030|GO:0005773

Gene79,GO:0009684|GO:0048523|GO:0022900|GO:0042742|GO:0009535|GO:0020037|GO:0009723|GO:0005783|GO:0005634

Gene80,GO:0009750|GO:0009941|GO:0016126|GO:0006833|GO:0016020|GO:0030243|GO:0015131|GO:0009651|GO:0015139|GO:0009624|GO:0009832|GO:0015367|GO:0016049|GO:0009534|GO:0019676|GO:0015742|GO:0006814|GO:0015729|GO:0071423|GO:0005739

Gene81,GO:0009750|GO:0043255|GO:0009611|GO:0048046|GO:0009805|GO:0006833|GO:0009825|GO:0004568|GO:0030247|GO:0006816|GO:0010075|GO:0009651|GO:0006032|GO:0007389|GO:0009809|GO:0042742|GO:0043481|GO:0048767|GO:0005794|GO:0006972|GO:0007030|GO:0010337|GO:0008361|GO:0006096|GO:0030244|GO:0019761|GO:0009926|GO:0010167|GO:0046686|GO:0009409|GO:0019344|GO:0009408|GO:0009737|GO:0009932|GO:0009735|GO:0016998|GO:0071555|GO:0009269

Gene82,GO:0009790|GO:0018454|GO:0004303|GO:0045703|GO:0042761|GO:0016020|GO:0005783|GO:0055114|GO:0000166
Gene83,GO:0009910|GO:0033523|GO:0009650|GO:0009965|GO:0010228|GO:0004842
Gene84,GO:0010103|GO:0005960|GO:0006098|GO:0010207|GO:0004375|GO:0016740|GO:0009507|GO:0019344|GO:0009657|GO:0006364|GO:0019464|GO:0005739
Gene85,GO:0010200|GO:0006499|GO:0006865
Gene86,GO:0010287|GO:0006979|GO:0046686|GO:0006096|GO:0004332|GO:0005739
Gene87,GO:0016020
Gene88,GO:0016020|GO:0005215
Gene89,GO:0016020|GO:0044464
Gene90,GO:0016021|GO:0005618|GO:0005739|GO:0009941|GO:0006810|GO:0005886
Gene91,GO:0016023|GO:0016020|GO:0055114
Gene92,GO:0016132|GO:0055085|GO:0009750|GO:0009414|GO:0009651|GO:0016021|GO:0009506|GO:0006084|GO:0015250|GO:0009266|GO:0010106|GO:0006972|GO:0006826|GO:0005515|GO:0006833|GO:0007030|GO:0006096|GO:0009507|GO:0046686|GO:0005886|GO:0016126
Gene93,GO:0016192|GO:0005829|GO:0006996|GO:0030132|GO:0030130|GO:0044765|GO:0006886|GO:0005198
Gene94,GO:0016491
Gene95,GO:0016579|GO:0016567|GO:0006511|GO:0004842
Gene96,GO:0016881|GO:0016567|GO:0009791|GO:0000166|GO:0048608
Gene97,GO:0019904
Gene98,GO:0032440|GO:0005524|GO:0006950|GO:0055114
Gene99,GO:0042742|GO:0046872|GO:0046688|GO:0000303|GO:0004784|GO:0010193|GO:0006801|GO:0010039|GO:0005829|GO:0005783|GO:0009651|GO:0055114
Gene100,GO:0043161|GO:0051510|GO:0043248|GO:0051049|GO:0051788|GO:0009960|GO:0004842|GO:0016567|GO:0005515|GO:0042023|GO:0005737|GO:0005634
Gene101,GO:0044237|GO:0016740
Gene102,GO:0044260|GO:0009941|GO:0030093|GO:0009508|GO:0006796|GO:0009773|GO:0044085|GO:0006139|GO:0009737|GO:0009657
Gene103,GO:0045158|GO:0022900|GO:0008121|GO:0051537|GO:0016021|GO:0042742|GO:0046872|GO:0010196|GO:0009941|GO:0009496|GO:0009535|GO:0015979|GO:0080167|GO:0005886
Gene104,GO:0045430|GO:0009744|GO:0010224|GO:0009718|GO:0009507|GO:0080167
Gene105,GO:0045454|GO:0009055|GO:0015035|GO:0006662
Gene106,GO:0045551|GO:0052747|GO:0009055|GO:0020037|GO:0004024|GO:0009809|GO:0008270|GO:0000166|GO:0022900|GO:0080167
Gene107,GO:0046939|GO:0004849|GO:0004127|GO:0005524|GO:0006222|GO:0005634|GO:0046899
Gene108,GO:0046961|GO:0008270|GO:0046933|GO:0009507|GO:0005618|GO:0005730|GO:0005754|GO:0015986
Gene109,GO:0048038|GO:0006511|GO:0008270|GO:0008137|GO:0005747|GO:0051788|GO:0080129|GO:0051539|GO:0006120|GO:0006486|GO:0009853
Gene110,GO:0048768|GO:0042742|GO:0001558|GO:0009579|GO:0009860|GO:0010252|GO:0048527|GO:0009507|GO:0008283|GO:0046686|GO:0090406|GO:0040014|GO:0005634|GO:0005886|GO:0007346|GO:0009790|GO:0048046

Gene111,GO:0051287|GO:0005507|GO:0005618|GO:0048316|GO:0055114|GO:0009744|GO:0008270|GO:0005730|GO:0009651|GO:0042742|GO:0009408|GO:0004365|GO:0006096|GO:0050661|GO:0005740|GO:0009507|GO:0046686|GO:0042542|GO:0005886|GO:0048046
Gene112,GO:0051287|GO:0005507|GO:0005777|GO:0006739|GO:0009651|GO:0042742|GO:0006099|GO:0004450|GO:0000287|GO:0006102|GO:0010043|GO:0046686|GO:0005886|GO:0048046
Gene113,GO:0051287|GO:0005507|GO:0055114|GO:0080022|GO:0008270|GO:0048658|GO:0009536|GO:0080144|GO:0009555|GO:0004365|GO:0050661|GO:0006006|GO:0016020
Gene114,GO:0051788|GO:0009853|GO:0006094|GO:0006635|GO:0035266|GO:0043161|GO:0008568|GO:0010078|GO:0010311|GO:0009651|GO:0009407|GO:0090351|GO:0048829|GO:0080129|GO:0051510|GO:0005829|GO:0060968|GO:0009965|GO:0009933|GO:0006096|GO:0042023|GO:0007292|GO:0048232|GO:0008540|GO:0046686|GO:0009408|GO:0008233|GO:0005886|GO:0005634|GO:0005524
Gene115,GO:0051788|GO:0046961|GO:0006098|GO:0009853|GO:0008270|GO:0006833|GO:0009651|GO:0046933|GO:0080129|GO:0006972|GO:0006511|GO:0007030|GO:0005754|GO:0006096|GO:0015986|GO:0046686|GO:0009060|GO:0009266
Gene116,GO:0055114|GO:0009414|GO:0005739|GO:0009651|GO:0009926|GO:0009941|GO:0009705|GO:0004427|GO:0048366|GO:0032440|GO:0010008|GO:0015992|GO:0009678|GO:0005886
Gene117,GO:0055114|GO:0016706
Gene118,GO:0080167|GO:0005576|GO:0009506|GO:0009505|GO:0007020
Gene119,GO:0097159|GO:1901363
Gene120,NA
Gene121,NA
Gene122,NA
Gene123,NA
Gene124,NA
Gene125,NA
Gene126,NA
Gene127,NA
Gene128,NA
Gene129,NA
Gene130,NA
