# Supplementary material for: Transcriptome sequencing of Pinus kesiya var. langbianensis and comparative analysis in the Pinus phylogeny
Source: BMC Genomics. 2018 Oct 3;19:725. doi: 10.1186/s12864-018-5127-6 (PMC6171231; doi:10.1186/s12864-018-5127-6)
Supplement: Supplementary file 4 — File S2. Sequences of resistance unigenes (PDF 14 kb) [file 12864_2018_5127_MOESM4_ESM.pdf]

>SEQ11_isotig03867
cgggcagagcttctgcaccgtgcagctgcattactgaaagaacacaagaatcccatagcagaatgtcttgtcaaggaaattgccaaacctgcaaaggatgc
tgttacagaggttgtaaggtctggagatctcatttcttactgcgctgaggagggagtgaggatccttggtgaaggcaaatttcttgtatcggatagtttccctgg
caatgatagaaacaaattgtgtctgtcctcaaagattcctctgggtgtcattttagctattcctccattcaactatcctgtaaatcttgctgtgtctaagattggtcct
gcccttattgctgggaattcaattgtgttgaagccgccaactcagggagccgtagcagccttgcacatggtacactgcttccatttggctggattcccaaagg
gccttatcagttgtgtcacaggaaagggatctgaaattggtgattttctcacaatgcatccgggtataggctgtattagtttcactgggggtgacactggtattgc
aatttccaaaaaggctggcatgattcctcttcagatggaattaggtggaaaagatgcctgtattatacttgaggacgctgatctggatctggcagcagccaatg
ttgccaagggaggcttttcttacagtggtcagaggtgtactgcggtcaaagtagtccttgtgatggaatcagttgcagatattattgtagagaaggtgaatgcc
aaaattgcaaagatgactgttggcccaccagaagaaaactgtgagatcaccccggttgtcacagaatcctctgcaaatttcattgagggtcttgttatggattc
caagcaaaaaggagccaaactctgccaggagtacaagagagagggtaaccttatatggcctcttttggtagaccacgtgaggcctgacatgagacttgctt
gggaagagccttttgggccagtcttgcccgtcattaggatcaactctgttgaagaaggcattcatcattgcaatgccagcaattttggactccagggatgtatat
tcacaaaagacatcaacaaagcaatcatgataagtgatgccatggaaactgga
>SEQ2_CL1082.Contig1_All
atggccggcaacggtgcttttggtgaacttcttgaaggagaggtgtttaaatattactacgatggcgagtggagagcgtcttcttcggggaaatcggtggcca
ttatcaatcctacgactaggaagacacagtacagggttcaagcttgtacgcaggaagaagtgaataaggttgtggactcttctagggttgctcaaaaggcatg
ggcaaagactccccttggaaacgggcagagcttctgcaccgtgcagctgcattactgaaagaacacaagaatcccatagcagaatgtcttgtcaaggaaa
ttgccaaacctgcaaaggatgctgttacagaggttgtaaggtctggagatctcatttcttactgcgctgaggagggagtgaggatccttggtgaaggcaaattt
cttgtatcggatagtttccctggcaatgatagaaacaaattgtgtctgtcctcaaagattcctctgggtgtcattttagctattcctccattcaactatcctgtaaatct
tgctgtgtctaagattggtcctgcccttattgctgggaattcaattgtgttgaagccgccaactcagggagccgtagcagccttgcacatggtacactgcttcca
tttggctggattcccaaagggccttatcagttgtgtcacaggaaagggatctgaaattggtgattttctcacaatgcatccgggtataggctgtattagtttcact
gggggtgacactggtattgcaatttccaaaaagtctggcatgattcctcttcagatggaattaggtggaaaagatgcctgtattatacttgaggacgctgatctg
gatctggcagcagccaatgttgccaagggaggcttttcttacagtggtcagaggtgtactgcggtcaaagtagtccttgtgatggaatcagttgcagatattatt
gtagagaaggtgaatgccaaaattgcaaagatgactgttggcccaccagaagaaaactgtgagatcaccccggttgtcacagaatcctctgcaaatttcatt
gagggtcttgttatggattccaagcaaaaaggagccaaactctgccaggagtacaagagagagggtaaccttatatggcctcttttggtagaccacgtgagg
cctgacatgagacttgcttgggaagagccttttgggccagtcttgcccgtcattaggatcaactctgttgaagaaggcattcatcattgcaatgccagcaatttt
ggactccagggatgtatattcacaaaagacatcaacaaagcaatcatgataagtgatgccatggaaactggaacagttcaaattaactctgcacctgcccgc
ggaccggatcattttcctttccagggtctgagagacagtggcattggttctcagggaatcacaaatagcattctcatgatgacgaaagtcaagagcactgtcat
caacttgcctactccttcgtttacaatgggt
>SEQ4_PITA_000000350-RA
atggtttatttaattattcctgtaggctgtattagtttcactgggggtgacactggtattgcaatttccaaaaaggctggcatgattcctcttcatatggaattaggtg
gaaaagatgcctgtattatacttgaggacgctgatctggatctggcagcagccaatgttgtcaagggaggcttttcttacagtggtcagaggtgtactgcggtc
gaagtagtccttctgatggaatcagttagaagg
>SEQ0_PUT-177a-Pinus_banksiana-8213
atggccggcaacggtgcttttggtgaacttcttgaaggagaggtgtttaaatattactatgatggagagtggagagcgtcttcttcggggaaatcagtggccat
tatcaatcctacgactaggaagacacagtacagggttcaagcttgtacgcaggaagaagtgaataaggttgtggactcttctagggttgctcaaaaggcatg
ggcaaagactccccttggaaacgggcagagcttctgcaccgtgcagctgcattactgaaagaacacaagaatcccatagcagaatgtcttgtcaaggaaa
ttgccaaacctgcaaaggatgctgttacagaggttgtaaggtctggagatctcatttcttactgcgctgaggagggagtgaggattcttggtgaagggaaattt
cttgtatcggatagtttccctggcaatgatagaaacaaattatgtctgtcctcaaagattcctctgggtgtcattttagctattcccccattcaactatcctgtaaatc
ttgctgtgtctaagattggtcctgcccttattgctgggaattcgattgtgttgaagccgccaactcagggagccgtagcagccttgcacatggtacactgcttcc
atttggctggattcccaaagggccttatcagttgtgtcacaggaaagggatctgaaattggtgattttctcacaatgcatccgggtataggctgtattagtttcact
gggggtgacactggtattgcaatttccaaaaaggctggcatgattcctcttcagatggaattaggtggaaaagatgcctgtattatacttgaggacgctgatct
ggatctggcagcagccaatgttgtcaagggaggcttttcttacagtggtcagaggtgtactgcggtcaaagtagtccttgtgatggaatcagttgcagatatta
ttgtagagaaggtgaatgccaaaattgcaaagatgactgttggtccaccagaagaaaactgtgagatcaccccggttgtcacagaatcctctgcaaatttcatt
gagggtcttgttatggattccaagcaaaaaggagccaaactctgccaggagtacaagagagagggtaaccttatttggcctcttttggtagaccacgtgagg
cctgacatgagacttgcttgggaagagccttttgggccagtcttgcccgtcattaggatcaactctgttgaagaaggcattcatcattgcaatgccagcaatttt
ggactccagggatgtatattcacaaaagacatcaacaaagcaatcatgataagtgatgccatggaaactggaacagtccaaattaactctgcacctgcccgc
ggaccggatcattttcctttccagggtctgagagacagtggcattggttctcagggaatcacaaatagcatcctcatgatgacgaaagtcaagagcactgtca
tcaacttgcctactccttcgtttacaatgggt
>SEQ2_CL1082.Contig1_All
atggccggcaacggtgcttttggtgaacttcttgaaggagaggtgtttaaatattactacgatggcgagtggagagcgtcttcttcggggaaatcggtggcca
ttatcaatcctacgactaggaagacacagtacagggttcaagcttgtacgcaggaagaagtgaataaggttgtggactcttctagggttgctcaaaaggcatg
ggcaaagactccccttggaaacgggcagagcttctgcaccgtgcagctgcattactgaaagaacacaagaatcccatagcagaatgtcttgtcaaggaaa
ttgccaaacctgcaaaggatgctgttacagaggttgtaaggtctggagatctcatttcttactgcgctgaggagggagtgaggatccttggtgaaggcaaattt
cttgtatcggatagtttccctggcaatgatagaaacaaattgtgtctgtcctcaaagattcctctgggtgtcattttagctattcctccattcaactatcctgtaaatct
tgctgtgtctaagattggtcctgcccttattgctgggaattcaattgtgttgaagccgccaactcagggagccgtagcagccttgcacatggtacactgcttcca
tttggctggattcccaaagggccttatcagttgtgtcacaggaaagggatctgaaattggtgattttctcacaatgcatccgggtataggctgtattagtttcact

gggggtgacactggtattgcaatttccaaaaagtctggcatgattcctcttcagatggaattaggtggaaaagatgcctgtattatacttgaggacgctgatctg
gatctggcagcagccaatgttgccaagggaggcttttcttacagtggtcagaggtgtactgcggtcaaagtagtccttgtgatggaatcagttgcagatattatt
gtagagaaggtgaatgccaaaattgcaaagatgactgttggcccaccagaagaaaactgtgagatcaccccggttgtcacagaatcctctgcaaatttcatt
gagggtcttgttatggattccaagcaaaaaggagccaaactctgccaggagtacaagagagagggtaaccttatatggcctcttttggtagaccacgtgagg
cctgacatgagacttgcttgggaagagccttttgggccagtcttgcccgtcattaggatcaactctgttgaagaaggcattcatcattgcaatgccagcaatttt
ggactccagggatgtatattcacaaaagacatcaacaaagcaatcatgataagtgatgccatggaaactggaacagttcaaattaactctgcacctgcccgc
ggaccggatcattttcctttccagggtctgagagacagtggcattggttctcagggaatcacaaatagcattctcatgatgacgaaagtcaagagcactgtcat
caacttgcctactccttcgtttacaatgggt
>SEQ3_PUT-175a-Pinus_contorta-5514
atggtacactgcttccatttggctggattcccaaagggccttatcagttgtgtcacaggaaagggatctgaaattggtgattttctcacaatgcatccgggtata
ggctgtattagtttcactgggggtgacactggtattgcaatttccaaaaaggctggcatgattcctcttcagatggaattaggtggaaaagatgcctgtattatac
ttgaggacgctgatctggatctggcagcagccaatgttgtcaagggaggcttttcttacagtggtcagaggtgtactgcggtcaaagtagtccttgtgatgga
atcagttgcagatattattgtagagaaggtgaatgccaaaattgcaaagatgactgttggtccaccagaagaaaactgtgagatcaccccggttgtcacagaa
tcctctgcaaatttcattgagggtcttgttatggattccaagcaaaaaggagccaaactctgccaggagtacaagagagagggtaaccttatttggcctcttttg
gtagaccacgtgaggcctgacatgagacttgcttgggaagagccttttgggccagtcttgcccgtcattaggatcaactctgttgaagaaggcattcatcattg
caatgccagcaattttggactccagggatgtatattcacaaaagacatcaacaaagcaatcatgataagtgatgccatggaaactggaacagttcaaattaac
tctgcacctgcccgcggaccggatcattttcctttccagggtctgagagacagtggcattggttctcagggaatcacaaatagcatcctcatgatgacgaaag
tcaagagcactgtcatcaacttgcctactccttcgtttacaatgggt
>SEQ6_PUT-187a-Pinus_sylvestris-52858
atgagatcgtctgcaggggacgcttcgaacatgccagaccctacggattcagtcgatgtactcaaacgcaaatttgctgcaaagggtttatctgcatcagatc
ttgttgttctcaatggagcacacacgatcggaacaactgcatgtttcttcatcgaggaccgtctgtacaacttcgcaggcgctggccacgcagacccctctatg
aatcccggatacttgagggaactgagatcaatttgcccccagggcggtgatgttaatgcgagggtggctctggacaggggttccgaattccaatttgacgac
gaattcctagaaaacgtgagggagggcaacggagcgctacagtctgatgcgagcatgtaccaagacagttccacccgcggatacatcgactcctattttgg
gctcctcggtggccttctggggccttcatttgaatctgactttgcagatgcaattgtaaagatggggcaggtcggcgtcaaaaccggttccaatggaaagattc
gtaccgtctgttcagtcctg
>SEQ2_Unigene23170_All
atgagatcctctgcaggggacgcttcgaacatgccagaccctacggattcagtcgatgtactcaaacgcaaatttgctgcaaagggtttatctgcatcagatct
tgttgttctcaatggagcacacacgatcggaacaactgcatgtttcttcatcgaggaccgtctgtacaacttcgcaggcgctggcggcgcagacccctctatg
aatcccggatacttgacggaactgagatcaatttgcccccagggcggtgatgttaatgcgagggtggctctggacaggggttccgaattccaatttgacgac
gaattcctagaaaacgtgagggagggcaacggagcgctacagtctgatgcgagcatgtaccaagacagttccacccgcggatacatcgactcctattttgg
gctcctcggtggccttctggggccttcatttgaatctgactttgcagatgcaattgtaaagatggggcaggtc
>SEQ5_isotig24007
atgagatcctctgcaggggacgcttcgaacatgccagaccctacggattcagtcgatgtcctcaaacgcaaatttgctgaaaagggtttatctgcatcagatct
tgttgttcttaatggagcacacacgatcggaacaactgcctgtttctttatcgaggaccgtctgtacaacttcgcagccgatggcgaggcagatccctctataa
atccgggatacttgacggaactgagatcaatttgccccaagggcggtgatgttaatgcgagggtggctctggacaggggttccgaattccaatttgacaacg
aattcctagaaaacgtgagggagggg
>SEQ1_comp65821_c3
atgagatcctctgcaggggacgcttcgaacatgccagaccctacggattcagtcgatgtcctcaaacgcaaatttgctgaaaagggtttatctgcatcagatct
tgttgttcttaatggagcacacacgatcggaacaactgcctgtttctttatcgaggaccgtctgtacaacttcgcagccgatggcgaggcagatccctctataa
atccgggatacttgacggaactgacatcaatttgccccaagggcggtgatgttaatgcgagggtggctctggacaggggttccgaattccaatttgacaacg
aattc
>SEQ12_isotig03955
atgggtgcagtggccaagcactcgaagggatggatatggtggtttttctcgttggccgtagcagcatttgtggtaacggcagcagtgttaacaatactacattc
caggaagcattctcaccatcaggtctcgccagttccaggtcctcccggagccatttctcagaaatatgcccaagccttgaagctcgcattgaccttctttcctat
tcagaaggctgggaagcttcctaatgataatcccataccatggagagggaactctgctctgtcagatggtaatgatgtgggtgcagacctttctggaggaatg
tatgatgcaggggatcacatcaaatttgggttacccatggcttttactgcaactcttttgtcttggggtgtgttggagtacggggatcagatggatgctgctcatc
aactaggggcagcccagtcttccatcagatggatcacagattatttgatcaacgcacatccttcaagcaatgtattgtatgttcaggtgggaaatgcaagcatt
gatcataagtgttgggaaaggcctgaaaatatgaatgagaaaaggccaactctcagggtagacgcttcaagtcctggatcggatgtagcagcagagacag
ctgcagcaatggcatctgcttcattagtgttccgcaatacagattctgcatactcagatatattgattcgtcacgcacaagaattgtttcagtttgctgactcttacc
gaggctcctatagcaatagcataccttcagttcaagccttctacaattctactggatataaagatgagcttctgtgggcagcagcctggctttatcatgcaaccc
acaatgaatcatacttgaactacgtttcagtagggaatggcgaagattttgcccaatggggagctgctccgacttggtttagctgggacaacaaacatgctgg
cgtgcaggttcttctttcgagagttcaatttttcgggacaaagactatgacttctgcagttcgttctgatcttgaacaatacaaaatgacagcagaagcaatcatct
gcgctcttcttcctcattcaccaacttcggctcccaatagaacagagggtggactcatatggataacacaatggaatgccatccaacattccgtaaattctgcct
tacttgcactgatatatagtgactacttgttgacgtcaagagttgagaacatgaaatgtggagacaaaaaagacttcactccacaggatttgcgcaagtttgcat
tttctcaggtggattatattttgggtgagaatccaatgaggatgagctatttggtaggctttggggataattacccaaaacaggtccaccatagaggtgcttccat
acctgctgatacgtccaaggtgtatgattgtaatagtggatatgtatggttcaattccaaatctccgaatccgaatgtggcaacaggggcattagttggaggac

cttttcagaatgatagttttattgatatgagatccaatcccatgcaaaatgaaccaagtacttacaatagtgctgctttggtaggcctcttatctggattactcacta
cttcttctgttgttcagtcttttatc
>SEQ0_PUT-177a-Pinus_banksiana-10254
ggggcatctgcttcattagtgttccgcaatacagattctgcatactcagatatattgattcgtcacgcacaagaattgtttcagtttgctgactcttaccgaggctc
ctatagcaatagcataccttcagttcaagccttctacaattctactggatataaagatgagcttctgtgggcagcagcctggctttatcatgcaacccacaatga
atcatacttgaactacgtttcagtagggaatggcgaagattttgcccaatggggagctgctccgacttggtttagctgggacaacaaacatgctggcgtgcag
gttcttctttcgagagttcaatttttcgggacaaagactatgacttctgcagttcgttctgatcttgaacaatacaaaatgacagcagaagcaatcatctgtgctctt
cttcctcattcaccaacttcggctcccaatagaacagagggtggactcatatggataacacaatggaatgccatccaacattccgtaaattctgccttacttgca
ctgatatatagtgactacttgttgacgtcaagagttgagaacatgaaatgtggagacaaaaaagacttcactccacaggatttgcgcaagtttgcattttctcag
gtggattatattttgggtgagaatccaatgaggatgagctatttggtaggctttggggataattacccaaaacacgtccaccatagaggtgcttccatacctgct
gatacgtccaaggtgtatgattgtaatagtggatatgtatggttcaattccaaatctccgaatccgaatgtggcaacaggggcattagttggaggaccttttcag
aatgatagttttattgatattagatccaatcccatgcaaaatgaaccaagtacttacaatagtgctgctttggtaggcctcttatctggattactcactacttcttctgt
tgttcagtcttttatc
>SEQ2_Unigene11406_All
atgaggttatcagggatcctcataattattttggttgtggctttggacgtggcagctggaatcttaggaatccatgcatacatggctcagaaccaggtgaggca
cctacatttatttgtatttgaatgcagagagcctagtcacgaagcattcaagctgggaatagcagctgcttcaatcttgttaattgcccacataattgccaacgta
gctgggggttgtatttgttatggatccagggatgacctcagaaattctccagtcaacaggcaaattgctgggatatctctcatgctttcatgggtcattttaggaa
tagcgtttggattactgatacttggagccatgtacaacaaccattccggggatcaatgttctctgagtcgccataagttcttgtggatgggaggagtactgtgctt
tgttcatgctgcaattattgctgcatattatattgcagccacttcaactatcatgctaaatagggatatcaaaggattgtccatggctgaaagaggaggaggg
>SEQ3_PUT-175a-Pinus_contorta-11400
atgaggttatcaggcatcctcataattattttggttgtggctttggacgtggccgctggaatcttaggaatccatgcatacacggctcagaaccaggtgaggca
actacatttatttgtaattgaatgcaaagagcctagtcacgaagcattcaagctgggaatagcagctgcttcactcttgttaattgcccacataattgccaacgta
gctgggggttgtatttgttatggatccagggatgacctcagaaattctccagtcaacaggcaaattgctgggatatctctcatgctttcatgggtcattttaggaa
tagcgtttggattactgctacttggagccaagtacaacaaccattccggggaacactgttctctgagtcaccataagttcttgtggatgggaggagtactgtgct
ttgttcatgctgcaattattgctgcatattatatttcagccacttcacctatcatgccaaatagggatatcaaaggactgtccatggctgaaagaggaggaggg
>SEQ4_PITA_000045763-RA
atgggtgcagtggccaagcactcgaagggatggatatggtggtttttctcgttggccgtagcagcatttgtggtaacggcagcagtgttaacaattctacattc
caggaagcattctcaccatcaggtctcgccagttccaggtcctcccggagccatttctcagaaatatgcccaagccttgaagctcgcattgaccttctttcctat
tcagaaggctgggaagcttgctaatgataatcccataccatggagagggaactctgctctgtcagatggtaatgatgtgggtgcagatctttctggaggaatg
tatgatgcaggggatcacatcaaatttgggttacccatggcttttactgcaactcttttgtcttggggtgtgttggagtacggggatcagatggatgctgctcatc
aactaggggcagcccagtcttccatcagatggatcacagattatttgatcaacgcacatccttcaagcaatgtattgtatgttcaggtgggaaatgcaagcatt
gatcataagtgttgggaaaggcctgaaaatatgaatgagaaaaggccaactctcagggtagacgcttcaagtcctggatcggatgtagcagcagagacag
ctgcagcaatggcatctgcttcattagtgttccgcaatacagattctgcatactcagatatattgattcgtcacgcacaagaattgtttcagtttgctgactcttacc
gaggctcctatagcaatagcataccttcagttcaagccttctacaattctactggatataaagatgagcttctgtgggcagcagcctggctttatcatgcaaccc
acaatgaatcatacttgaactacgtttcagtagggaatggcgaagattttgcccaatggggagctgctccgacttggtttagctgggacaacaaacatgctgg
cgtgcaggttcttctttcgagagttcaatttttcgggacaaagactatgacttctgcagttcgttctgatcttgaacaatacaaaatgacagcagaagcaatcatct
gtgctcttcttcctcattcaccaacttcggctcccaatagaacagagggtggactcatatggataacacaatggaatgccatccaacattccgtaaattctgcct
tacttgcactgatatatagtgactacttgttgacgtcaagagttgagaacatgaaatgtggagacaaaaaagacttcactccacaggatttgcgcaagtttgcat
tttctcaggtggattatattttgggtgagaatccaatgaggatgagctatttggtaggctttggggataattacccaaaacacgtccaccatagaggtgcttccat
acctgctgatacgtccaacgtgtatgattgtaatagtggatatgtatggttcaattccaaatctccgaatccgaatgtggcaacaggggcattagttggaggac
cttttcagaatgatagttttattgatattagatccaatcccatgcaaaatgaaccaagtacttacaatagtgctgctttggtaggcctcttatctggattactcactac
ttcttctgttgttcagtcttttatc
>SEQ7_isotig05514
atgaggttatcagggatcctcataattattttggttgtggctttggacgtggcggctggaatcttaggaatccatgcatacatggctcagaaccaggtgaggca
cctacggttatttttatttgaatgcagagagcctagtcacgaagcattcaagctgggaatagcagctgcttcaatcttgttaattgcccacataactgccaacgta
gctgggggttgtatttgttatggatccagggatgagctcagaaattctccagtcaacaggcaaattgctgggatatctctcatgctttcatgggtcattttaggaa
tagcgtttgcattactggtgcttggagccatgtccaacaaccattccagggattactgttctctgagtcgccataagttcttgtggatggggggagtactgtgctt
tgttcatgctgcaattattgctgcatattatattgcagccacttcaactaacatgctaaatagggatatcaaaggactgtccatggctgaaagaggc
>SEQ8_comp18337_c1
atgtccaacaaccattccagggattactgttctctgagtcgccataagttcttgtggatgggaggagtactgtgctttgttcatgctgcaattattgctgcatattat
attgcagccacttcaactatcatgctaaatagggatatcaaaggactgtccatggctgaaagaggagggggg
